# Subconcussive Head Injuries Negatively Affect Academic Achievement in Adolescent Males

**DOI:** 10.3390/children13030399

**Published:** 2026-03-13

**Authors:** Michael A. Carron, Lauren E. Caplick, Vincent J. Dalbo

**Affiliations:** 1 Health, Education, Lifestyle, and Performance Laboratory, St Brendan’s College, Yeppoon, QLD 4703, Australia; m.carron@cqu.edu.au (M.A.C.);; 2 School of Health, Medical and Applied Sciences, Central Queensland University, Rockhampton, QLD 4702, Australia

**Keywords:** concussion, child, traumatic brain injury, education, recovery, school

## Abstract

**Highlights:**

**What are the main findings?**

- The occurrence of a subconcussive head injury resulted in a meaningful and significant reduction in GPA during the term in which the head injury occurred.
- We found students to have diminished academic performance with 26.93 ± 15.22 days of recovery following a subconcussive head injury.

**What are the implications of the main finding?**

- Our findings provide initial evidence suggesting the need for subconcussive head injuries to be included in future return to learn protocols.
- Our findings provide support for subconcussive head injuries to be officially recognised as an injury resulting in acute cognitive impairment.

**Abstract:**

**Background/Objectives**: To determine the effects of a subconcussive head injury on adolescent student academic achievement assessed by grade point average (GPA). **Methods**: The study utilised an experimental (subconcussive head injury, *n* = 45) and a matched pair control group (*n* = 45). Data were collated at baseline (i.e., the term prior to sustaining a subconcussive head injury) and the term the subconcussive head injury occurred. Subconcussive head injuries were preliminarily assessed onsite by a registered nurse and diagnosed by a general practitioner using established protocol. The average subconcussive head injury occurred 26.93 ± 15.22 days prior to the exam period, which is when all graded assessments/examinations occurred. All participants (*N* = 90) were adolescent males (age: 14.04 ± 1.48 years) in grades 7–12 (grade: 8.62 ± 1.51). An independent t-test was used to test for potential between group differences at baseline. Separate dependent t-tests were used to test for the effects of a subconcussive head injury on GPA in the experimental group and the effects of time on GPA in the control group. Standardised Cohen’s *d* with 95% confidence intervals were used to quantify the meaningfulness of the potential between or within group differences. **Results**: Non-meaningful, non-significant differences were revealed for all variables between the experimental and control group at baseline. A subconcussive head injury resulted in a meaningful and significant decrease in GPA (*d* = −0.417, 95% CI = −0.720 to −0.110, *small*, *p* = 0.008); while a non-meaningful, non-significant increase in GPA occurred in the matched pair control group (*d* = 0.037, 95% CI = −0.256 to 0.329, *trivial*, *p* = 0.808). **Conclusions**: Our findings provide initial evidence suggesting the need for return to learn protocols to consider subconcussive head injuries.

## 1. Introduction

Head injuries are defined as an injury to the skull, scalp, or brain resulting from an external mechanical force [1] and led to 406,000 emergency department presentations and 142,000 hospitalisations between 2020 and 2021 in Australia [2]. During this same period of time, 1 in 50 Australian children and adolescents between the age of 5 and 14 years were admitted to an emergency department due to a head injury [2]. In fact, head and neck injuries were a common injury site in Australian children and adolescents (≤18 years) presenting to an emergency department and accounted for 21% of emergency department presentations and hospitalisations between 2020 and 2021 [2]. Between 2023 and 2024, sports related head and neck injuries accounted for approximately 11,100 hospital cases [3].

**Figure 1:**
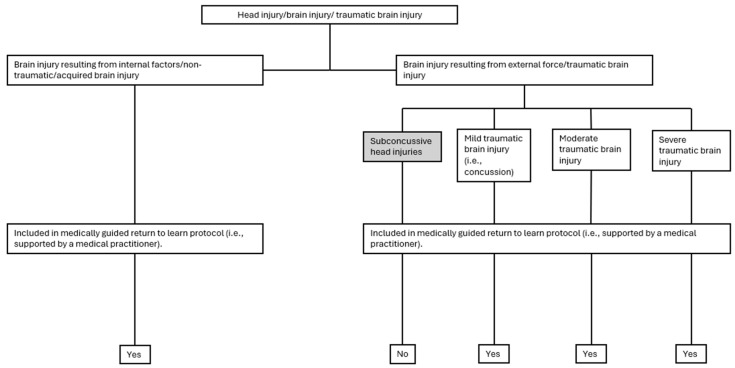
Schematic of current classification of head injuries. Note: Grey indicates a type of head injury that does not have an official classification.

The term head injury is commonly used synonymously with the terms brain injury and/or traumatic brain injury (TBI) [4]. This practice is less than ideal as an individual with a head injury may or may not present with a brain injury. Moreover, a non-traumatic/acquired brain injury refers to a brain injury resulting from internal factors such as lack of oxygen, exposure to toxins, or a tumour [5,6]. On the other hand, a traumatic brain injury refers to a brain injury resulting from an external force [5,7]. In this regard, traumatic brain injuries range from mild to severe [8], and a concussion refers to symptoms meeting the classification of a mild traumatic brain injury [9], which is typically diagnosed using an assessment tool such as, but not limited to, the Glasgow Coma Scale or Sport Concussion Assessment Tool, 5th Edition (SCAT5) [10]. Confusingly, there is currently no established classification of traumatic brain injury less severe than a mild traumatic brain injury (i.e., concussion) but traumatic brain injuries of this type are often referred to as head injuries or subconcussive head injuries (Figure 1). Importantly, subconcussive head injuries may result in symptoms that overlap with a mild traumatic brain injury (i.e., a concussion) but do not meet the clinical diagnosis of this injury [11], which is problematic given the current return to learn protocol utilised in schools only provide recommendations for individuals diagnosed with a mild traumatic brain injury [12].

Implementing a return to learn protocol that applies to mild traumatic brain injuries (i.e., concussion) [12] or those greater in severity overlooks the broader spectrum of traumatic head injuries that do not meet this classification yet impair cognitive function (i.e., subconcussive head injuries). For example, while a subconcussive traumatic head injury is less severe than a mild traumatic brain injury, each injury involves an impact to the head that may result in a brain injury and the presentation of symptoms such as acute cognitive impairment (i.e., slowed processing speed) [11], diminished memory, impaired problem-solving ability, and poor sleep [13]. In this regard, Tsushima et al., [14] investigated subclinical head injuries (i.e., subconcussive head injuries) among 641 non-concussed school athletes (464 males, 177 females) using results from an Immediate Post-Concussion Assessment and Cognitive Testing (ImPact) evaluation administered online. The authors [14] compared the effects of repetitive head trauma on GPA by examining a high contact group (males: *n* = 412, females: *n* = 41, mean age of combined group: 15.20 ± 1.22 years, sports: American Football and wrestling/martial arts) and a low contact group (males: *n* = 52, females: *n* = 136, mean age of combined group: 14.88 ± 1.22 years, sports: baseball and volleyball). The authors [14] reported, athletes in the high impact group who sustained repetitive head trauma resulting in non-concussive head injuries performed significantly poorer in GPA, visual motor speed, and reaction time compared to athletes in the low contact group. However, the findings assume the frequencies of subconcussive head injuries were different between the sports (i.e., the high and low contact groups) [14].

To the authors’ knowledge, no known research has examined the effects of a diagnosed subconcussive head injury on GPA during the term in which the injury occurred. Such information is critical for demonstrating if adolescents who experience a subconcussive head injury should be included in future return to learn protocols. Given subconcussive head injuries may result in symptoms that impair learning, we hypothesise the occurrence of a subconcussive head injury will negatively affect the academic performance of adolescence and will be reflected by a decrease in GPA. If our supposition is correct, our findings would provide evidence for future return to learn protocols to include subconcussive head injuries in their framework. Our findings would also provide support for subconcussive head injuries to be officially recognised as a diagnosed traumatic head injury.

## 2. Materials and Methods

### 2.1. Participants

Data were retrospectively collected from the same independent, religiously affiliated, all male high school from January 2021 to September 2024. Participants ranged in age from 12 to 18 years old (age: 14.04 ± 1.48 years) and ranged from 7th to 12th grade (grade: 8.62 ± 1.51). In Australia, a grade refers to year level corresponding to the progression of a student through the education system. High school is typically comprised of grades 7 to 12. Participants were split into an experimental group and a matched pair control group. Exclusion criteria for the experimental group consisted of the following: (1) Not being diagnosed with a subconcussive head injury. (2) Being diagnosed with a mild traumatic brain injury (i.e., concussion). (3) Being diagnosed with a severe traumatic brain injury. (4) If diagnosed with multiple subconcussive head injuries the potential participant would be excluded if the second subconcussive head injury occurred during the term immediately following the term of the first subconcussive head injury. (5) Diagnosed with a subconcussive head injury during the exam period as the student could have completed exams prior and following the subconcussive head injury. The only inclusion criterion for the experimental group was the participant sustained a head injury that did not meet the diagnoses of a mild traumatic brain injury or a severe traumatic brain injury initially assessed by an onsite health professional (i.e., one of the registered nurses employed by the high school) and diagnosed by a general practitioner.

Potential participants in the matched pair control group were excluded if they were diagnosed with a subconcussive head injury, mild traumatic head injury, or severe traumatic head injury by an onsite health professional during the time period monitored in the study. Matched pair control group participants were selected on their likeness to a specific participant in the experimental group at baseline (i.e., the term prior to the occurrence of a subconcussive head injury). Specifically, matched pair control participants were selected on the following characteristics: school, sex, grade, term of the subconcussive head injury (i.e., if a subconcussive head injury occurred in a participant during grade 8, term 2 in 2024, the two timepoints would be grade 8, term 1 and grade 8, term 2 2024 for the participant in the experimental group and the matched pair control participant), and GPA. In instances when more than one participant was able to serve as a matched pair control, the first participant was always chosen.

**Figure 2:**
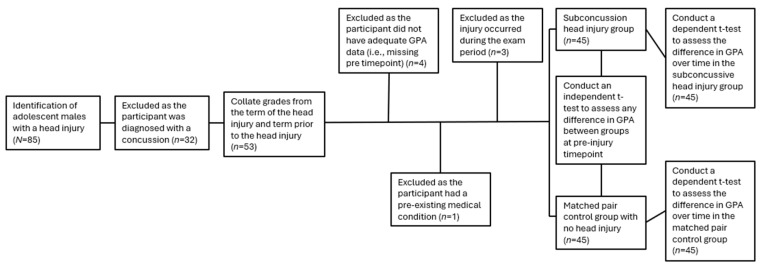
Schematic of study design. Note: Separate independent t-tests and standardised Cohen’s *d* assessments were conducted to demonstrate there were non-significant and non-meaningful differences between the experimental and matched control groups at baseline. The pre vs. post dependent t-test and standardised Cohen’s *d* assessment in the experimental group represent the effect of a subconcussive head injury on GPA. The pre vs. post dependent t-test and standardised Cohen’s *d* assessment of the matched pair control group represent the effect of time on GPA.

Data collation revealed 85 students incurred a traumatic head injury and visited the onsite health professional. Of the 85 students diagnosed with a traumatic head injury, 32 were classified as a mild traumatic brain injury (i.e., concussion) and those students were excluded from the study. Each of the remaining 53 students were diagnosed with a subconcussive head injury and were eligible for inclusion. We found 2 of the remaining students to be diagnosed with two subconcussive head injuries during the data collection period. In both instances, the subconcussive head injuries occurred ≥3 terms apart and as a result each student was included in the study. Specifically, data from the occurrence of their first subconcussive head injury were included in the statistical analysis. We found 3 of the remaining students incurred a subconcussive head injury during the exam period and they were excluded from the analysis. Of the remaining 50 students, 4 were excluded due to having no data for the term prior to that which they had sustained the head injury (e.g., year 7, term 1 students did not have data from previous term). An additional student was excluded due to a pre-existing medical condition (i.e., prior diagnoses resulting in head pain and was not subject to a traumatic head injury). As a result, 45 participants were included in the study (Figure 2). This study was conducted in accordance with the ethical principles outlined in the Declaration of Helsinki and retrospective approval was attained from a Human Research Ethics Committee (#0000025124).

### 2.2. Head Injury Diagnosis

Head injuries that occurred on school grounds that did not require an emergency hospitalisation were assessed onsite by a registered nurse employed by the high school. Students who sustained an offsite head injury that did not require an emergency room visit were also assessed by an onsite registered nurse if the student presented to the onsite health centre (i.e., an onsite centre where qualified nursing professionals see students). The onsite assessment consisted of a preliminary neurological screening, the SCAT 5 assessment in accordance with international concussion consensus guidelines, and an evaluation of symptoms. The assessments were completed in a quiet environment (i.e., onsite health centre) free from distractions. Results were recorded on standardised forms and stored securely onsite. Upon completion of the preliminary assessment, all students with a head injury and/or traumatic brain injury were referred to a general practitioner for formal diagnosis of a concussion or subconcussion (i.e., did not meet the criteria for concussion).

Currently, there is no validated diagnostic framework for subconcussive head injuries. As a result, the present study applied an operational, study specific definition which defined a subconcussive head injury as a traumatic head injury that did not meet the clinical criteria for a mild traumatic brain injury at the time of assessment and did not require emergency medical care, but warranted clinical evaluation by a general practitioner. Consequently, traumatic head injuries that were examined by a medical professional and did not meet the clinical criteria for a mild traumatic brain injury were classified as subconcussive head injuries. Students diagnosed with a subconcussive head injury returned to normal activities (i.e., no restrictions) following medical assessment. Students diagnosed with a mild traumatic brain injury (i.e., concussion) were excluded from the study and entered the medically guided return to learn protocol. No students were diagnosed with a severe brain injury.

### 2.3. Grade Point Average (GPA)

The GPA for all participants in our study was determined by the results of the end of term assessment and exam period which occurred during the last two weeks of each term. Teachers were responsible for grading the assessments and examinations that occurred during the exam period. Student raw GPA was quantified as follows: A+ = 14.01–15, A = 13.01–14, A− = 12.01–13, B+ = 11.01–12, B = 10.01–11, B− = 9.01–10, C+ = 8.01–9, C = 7.01–8, C− = 6.01–7, D+ = 5.01–6, D = 4.01–5, D− = 3.01–4, E+ = 2.01–3, E = 1.01–2, E− = ≤1. All classes were included and were equally weighted in the calculation of GPA. As a result, the GPA at the end of each term was quantified as: (the sum of the grade awarded for each subject completed during the term)/total number of subjects completed for the term.

### 2.4. Testing Timepoints

Data were collected at two timepoints for each participant in the experimental and matched pair control group. The timepoints correspond to periods when students receive their GPA for each school term. A school term is a standard subdivision of the school year typically lasting 9–11 weeks. In Australia, a school year is typically divided into four terms. The first timepoint was the term immediately prior to the term a participant in the experimental group sustained a subconcussive head injury and was utilised to establish a baseline of academic performance (i.e., GPA). The second timepoint was the term the subconcussive head injury occurred in the experimental group and was utilised to quantify the effect of experiencing a subconcussive head injury on academic performance (i.e., GPA). The matched pair control group was included in the study to account for a possible time effect on academic performance. Specifically, it was possible that GPA could decrease over time in the subconcussive head injury group due to classes becoming more difficult over time. Therefore, the matched pair control group was employed to quantify the effect of time on academic performance (i.e., GPA).

### 2.5. Statistical Analysis

Our preference would have been to analyse our results using a 2 × 2 RM ANOVA if our sample was adequately powered but our *a priori* power analysis using G*Power (3.1.9.7, G*Power) [15,16] with a *medium* effect size (*d* = 0.50), alpha level (*α*) of 0.05, and statistical power (*β*) at 80% revealed 128 participants (i.e., 64 per group) would have required to adequately power the group effect of the 2 × 2 RM ANOVA. However, our *a priori* power analysis for a dependent t-test with a *medium* effect size (*d* = 0.50), alpha level (*α*) of 0.05, and statistical power (*β*) at 80% yielded a result of 34 participants per group to be adequately powered. As a result, our experimental group was used to quantify the effects of a subconcussive head injury on GPA and our matched pair control group was used to quantify the effects of time on GPA.

Separate independent t-tests were conducted to demonstrate the matched pair control was not significantly or meaningfully different from the subconcussive head injury group at baseline. Results from our independent t-test revealed our data to demonstrate normality for GPA (Shapiro–Wilk: *p* > 0.05) but age, year group, and term of injury were non-normally distributed at baseline (Shapiro–Wilk: *p* < 0.001). However, this outcome was expected as the matched pair control group was created to match the subconcussive head injury group. Moreover, homogeneity of variance for each dependent variable was met (Levene’s tests: *p* > 0.05). Given independent t-tests are robust to violations of assumptions [17] paired with the expected similarity in data at baseline, parametric statistics were utilised to analyse the data. Separate dependent t-tests were used to quantify the effects of a subconcussive head injury on GPA in the experimental group (Shapiro–Wilk: *p* > 0.05) and the effects of time on GPA in the matched pair control group (Shapiro–Wilk: *p* = 0.017). Given dependent t-tests are robust to violations of assumptions [18], having an adequate sample size and visual inspection of the Q-Q plot for the comparison of GPA pre to GPA post in the matched pair control group appearing to be approximately normally distributed, parametric statistics were utilised to analyse the data.

Prior to data collection it was determined each statistical analysis would report the repeatability and meaningfulness of the finding. Repeatability was determined with significance and an *a priori* alpha-level of ≤0.05 was determined as needing to be met for a finding to be considered repeatable. Meaningfulness was determined utilizing an effect size and 95% confidence interval. For the independent and dependent t-tests standardised Cohen’s *d* was selected as the effect size measure and the direction of the effect size was reported as subconcussive head injury group—matched pair control group for the independent t-test, and post subconcussive head injury—pre subconcussive head injury for each dependent t-test. The effect size magnitude was determined based on the effect size and 95% confidence interval. Effect size magnitudes were determined as: *trivial* <0.20, *small* ≥0.20 to <0.50; *medium* ≥0.50 to <0.80, or *large* ≥0.80 [19]; however, if the 95% confidence interval contained 0, the effect size magnitude was determined to be *trivial* (i.e., non-meaningful). Since parametric statistics were utilised to analyse the results, data are presented as mean ± standard deviation (SD). All statistical analyses were conducted using Jamovi (Jamovi software; version 2.3.28; the Jamovi project, Sydney, Australia).

## 3. Results

**Figure 3:**
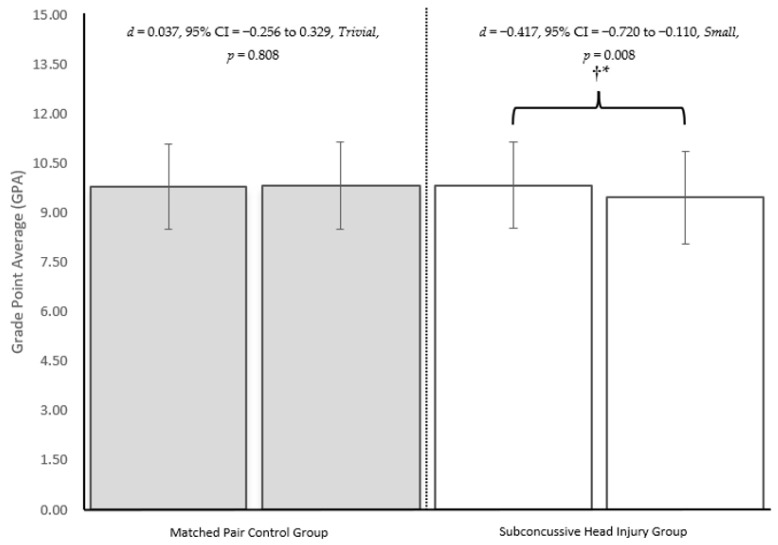
Results from separate dependent t-tests to assess the effects of time on GPA in the matched pair control group and the effects of a subconcussive head injury on GPA in the experimental group. Note: *d* = standardised Cohen’s *d*; CI = confidence interval; † = a meaningful effect; * = a significant effect. Meaningfulness reflects the magnitude of the effect, which is deemed as *trivial* if the 95% confidence interval overlaps zero. Significance reflects the repeatability of the finding.

The independent t-test revealed a non-meaningful, non-significant difference between the subconcussive head injury group and the matched pair control group regarding GPA, age, year group at school, and term of injury (Table 1). Subconcussive head injuries were found to occur 26.93 ± 15.22 days (range: 2 to 52 days) prior to the start of the two-week examination period. The dependent t-test of the matched pair control group revealed time to have a non-meaningful, non-significant effect on GPA. The dependent t-test in the experimental group revealed the incurrence of a subconcussive head injury to result in a meaningful, significant decrease in GPA (Figure 3).

## 4. Discussion

The negative long-term consequences of traumatic brain injuries are beginning to be better understood but research examining the effects of traumatic brain injuries in adolescents is still in its infancy, particularly regarding the effects of experiencing a subconcussive traumatic head injury on academic achievement (i.e., GPA). In fact, most research in this realm has examined the effects of mild traumatic brain injuries (i.e., concussion) on GPA and has been used to inform the return to learn protocol employed by schools to reintegrate students who experienced a mild traumatic brain injury back into an assisted learning environment. Concernedly, subconcussive head injuries may result in symptoms that overlap with concussion and can negatively impact learning such as acute cognitive impairment (i.e., slower processing), diminished memory, impaired thinking, and poor sleep [11,13]. However, subconcussive head injuries are not included in the current return to learn protocol. As a result, we quantified the effects of incurring a subconcussive head injury on academic performance in adolescent males and found the occurrence of a subconcussive head injury to meaningfully and significantly reduce GPA in the term the subconcussive head injury occurred. Results from our study provide preliminary evidence to suggest subconcussive head injuries should receive an official classification on the continuum of diagnosed traumatic brain injuries. Further, our findings provide initial evidence to suggest students diagnosed with a subconcussive head injury may benefit from being considered in future return to learn protocol policies, particularly if others are able to replicate our findings.

The strongest study design to employ to answer our research question would have been a 2 × 2 RM ANOVA but the main effect for group would have been underpowered with our sample size. As a result, a non-meaningful or non-significant between group differences at a timepoint could have been the result of having an increased probability of making a type II error (i.e., reporting no differences between the groups when a difference is present). However, our sample size was adequately powered to conduct dependent t-tests. As a result, we created an experimental group to quantify the effects of obtaining a subconcussive head injury on GPA and a matched pair control group to quantify the effects of time on GPA. We utilised this study design to ensure if a meaningful and/or significant decrease in GPA occurred in the subconcussive head injury group, the difference was not the result of a time effect (i.e., GPA decreasing due to classes becoming more difficult with an increase in age). Given there were no meaningful or significant differences between our experimental and control group at baseline we are confident the experimental group represents the effects of a subconcussive head injury on GPA and the matched pair control group represents the effects of time on GPA.

The matched pair control group experienced a non-meaningful, non-significant increase in GPA from baseline, meaning GPA was stable over time which is consistent with previous research findings [20,21]. Conversely, we found the incurrence of a subconcussive head injury to result in a meaningful, significant reduction in GPA providing initial evidence for subconcussive head injuries to be included in the return to learn protocol. Interestingly, the current return to learn protocol suggests 24 to 48 h of rest is required following the diagnosis of a mild traumatic head injury. It is questionable that there is not a set rest period with the initial rest prescribed varying from 24 to 48 h [12,22]. However, wherever recovery guidance is unclear concerning the required rest from an injury, it would be advisable to make the conservative recommendation of 48 h, as returning too early would likely result in an increased probability of negative outcomes compared to resting an additional 24 h.

Following the initial rest period, individuals progress though the remaining stages of the return to learn protocol starting with performing minor cognitive activity at home, followed by moderate cognitive activity at home, partial school attendance, gradual reintegration to school, and full mental workload (i.e., full-time school with no restrictions) [23]. No requisite time period is placed on any stage, but individuals can only progress through a maximum of one stage per day. Taking this into account, a systematic review and meta-analysis [24] of clinical recovery from concussion in returning to school and sport reported adolescent athletes on average returned to unrestricted learning 8.3 days (95% CI = 5.6 to 11.1 days) following a mild traumatic brain injury; with 93% of the sample returning to unrestricted learning after 10 days [24]. This finding is concerning as research has reported concussive symptoms resulting from a mild traumatic brain injury to persist for an average of 15 days in adolescents [25] with more recent research suggesting it may take >12 weeks (i.e., >84 days) for children and adolescents who experienced a mild traumatic brain injury to return to an acceptable level of functioning (i.e., optimal) across cognitive, physical, resilience, and socioemotional domains [26]. Taken together, these findings [24,25,26] suggest that despite the return to learn protocol being specifically designed to enable children and adolescents to return to unrestricted learning without deleterious effects following an incurrence of a mild traumatic brain injury students appear to be returning to unassisted learning while their ability to learn is impaired.

To further our point, we examined the effects of a subconcussive head injury on GPA, which is a less severe head injury than a mild traumatic brain injury. Yet, we found GPA to be negatively impacted by the incurrence of a subconcussive head injury, and in our study the diagnosed subconcussive head injury occurred 26.93 ± 15.22 days prior to the start of a two week examination period. Given most symptoms following a mild traumatic brain injury (i.e., concussion) have been reported to persist for an average of 15 days in adolescents [25] logic would imply GPA should not have been influenced by the subconcussive head injury in adolescents in our study. However, results from our study suggest physical symptoms (i.e., dizziness, balance issue, vision issues), environment tolerances (i.e., ability to handle social interactions and brightness/visual light exposure), as well as emotional and behavioural changes (i.e., anxiety, agitation) caused by the result of a traumatic brain injury may recover more quickly than cognitive functioning (i.e., ability to concentrate, remember information, the ability to process information). Taken as a whole, strong consideration should be given to include subconcussive head injuries to the return to learn protocol if our findings can be replicated and the structure of the return to learn protocol should be examined. Specifically, the current return to learn protocol appears to place too much responsibility of when to return to unrestricted learning on the individual who experienced a mild traumatic brain injury, and they may be making the decision to return to unassisted learning while still being cognitively impaired. However, due to the paucity of research it is not possible to currently prescribe an accurate minimum timeline to fully cognitively recover from a traumatic head injury.

Most research we found has examined the long-term (≥12 months) effects of a student experiencing a mild traumatic brain injury (i.e., concussion) on GPA [27,28,29]. In this regard, Tsushima et al. [28] compared the cumulative GPA of students who experienced a concussion during the school year (*n* = 39) to students who did not experience a concussion during the school year (*n* = 41) and concluded experiencing a concussion had no effect on GPA or neurocognitive test scores using the ImPACT assessment. In contrast, Lowry et al., [27] utilised data from the 2017 Youth Risk Behavior Survey to conclude students who self-reported experiencing a concussion to negatively impact their self-reported grades over a 12 month time period. While, Lystad et al., [29] examined the impact of concussion on objective school performance measures among students 7 to 18 years of age utilising National Assessment Program—Literacy and Numeracy (NAPLAN) [29] results and school graduation data. The authors [29] reported students who incurred a concussion had an increased risk of not attaining the national minimum standards for literacy and numeracy, and in senior years (i.e., 10, 11, and 12) were at greater risk of not completing high school. Combined, these studies [27,28,29] offer valuable information concerning the long-term objective and subjective effects of concussion; however, these previous studies [27,28,29] offer no information regarding the acute effect of a subconcussive or mild traumatic brain injury on GPA. Given the symptoms of subconcussive head injuries and mild traumatic brain injuries can negatively impact learning and resolve in approximately 15 days [25], it is logical the negative impacts of a traumatic brain injury are going to be most pronounced around the acute phase of the injury. As a result, more research is required to better understand the acute effects of traumatic head injuries (i.e., subconcussive traumatic head injuries and mild traumatic brain injuries) on learning to inform best practice in the development of return to learn protocols.

### 4.1. Limitations

Although our study provides novel findings regarding the impact of a subconcussive head injury on academic performance our work can be built upon as our study only included males aged between 12–18 years (i.e., grades 7–12) from a single high school. We are also unaware of the previous history of head injuries of each participant. Specifically, it is possible the academic performance of participants with a history of traumatic head injuries were more negatively affected than participants who experienced their first traumatic head injury, but we only had access to health records for each participant dating back to 2022. Moreover, there was no formal cognitive assessment, nor measures of mental health status or the recovery trajectories of students within the present study which may influence recovery from a subconcussive head injury. Consequently, we recommend future research systematically monitor changes in mental health and recovery trajectories to determine their influence on academic outcomes. Finally, although all head injuries were initially screened by a nurse and diagnosed by a general practitioner, a subjective measure of the severity of the head injury to the participant was not obtained, nor were any symptoms of the traumatic head injury or cause of the injury (e.g., sport or recreational). If monitored across a large sample, tracking the severity and symptom burdens of the traumatic head injury could allow for the use of more advanced data analyses.

### 4.2. Practical Implications

Our work yielded several meaningful outcomes. First, subconcussive traumatic head injuries are not currently recognised in return to learn protocols. However, results from our work provide initial support for subconcussive head injuries to be recognised as an official classification of traumatic brain injury as we found subconcussive head injuries to negatively impact performance in adolescent males. Second, our findings provide initial evidence to suggest subconcussive head injuries should be included in the current return to learn protocol to ensure students receive the necessary support and recovery time to perform to their true ability on assessments, tests, and exams. Third, children and adolescents following the current return to learn protocol have been reported to return to learning 8 days following a mild traumatic brain injury. We found students to have diminished academic performance with 26.93 ± 15.22 days of recovery following a subconcussive head injury. Together, these findings suggest children and adolescence following the current return to learn protocol are likely returning to unrestricted learning while still being cognitively impaired. Fourth, the current return to learn protocol seems to place too much emphasis on the individual who experienced the traumatic head injury regarding when to return to unassisted learning. As a result, future return to learn protocols should seek to provide at least some form of timeline for each return to learn step to help ensure individuals do not progress prematurely through the process.

## 5. Conclusions

Subconcussive head injuries are not presently recognised as an acute brain injury, nor considered within the return to learn guidelines; however, our findings provide evidence that a subconcussive traumatic head injury results in deleterious acute effects on GPA in adolescent male students. Accordingly, subconcussive head injuries should be recognised as an injury resulting in acute cognitive impairment; as such, students should be afforded support and adequate recovery time to better safeguard academic outcomes by being considered in return to learn protocols to reduce the risk of a premature return to learning.

## Figures and Tables

**Table 1:** Independent t-test comparison of the subconcussive head injury group and the matched pair control group at baseline.

Variable at Baseline	Matched-Pair Control Group (*n* = 45)	Subconcussive Head Injury Group (*n* = 45)	Standardised *d*, (95% CI)	Effect Size Magnitude	*p*-Value
GPA	9.79 ± 1.28	9.82 ± 1.31	−0.028 (−0.441 to 0.385)	*Trivial*	0.895
Age	14.04 ± 1.48	14.03 ± 1.50	−0.005 (−0.418 to 0.408)	*Trivial*	0.981
Year Group (Grade)	8.62 ± 1.51	8.62 ± 1.51	0.000 (−0.413 to 0.413)	*Trivial*	1.000
Term of Injury	2.00 ± 0.85	2.00 ± 0.85	0.000 (−0.413 to 0.413)	*Trivial*	1.000
Same school (Y/N)	Y	Y	-	-	-
Sex	Male	Male	-	-	-

Note: Meaningfulness reflects the magnitude of the effect, which is deemed as *trivial* if the 95% confidence interval overlaps zero. Significance reflects the repeatability of the finding.

## Data Availability

The original contributions presented in this study are included in the article. Further inquiries can be directed to the corresponding author.
